# Physiotherapy and Physical Activity as Factors Improving the Psychological State of Patients With Cancer

**DOI:** 10.3389/fpsyt.2021.772694

**Published:** 2021-11-22

**Authors:** Ewelina Zyzniewska-Banaszak, Jolanta Kucharska-Mazur, Aleksandra Mazur

**Affiliations:** ^1^Department of Rehabilitation Musculoskeletal System, Pomeranian Medical University, Szczecin, Poland; ^2^Department of Psychiatry, Pomeranian Medical University, Szczecin, Poland; ^3^Department of Social Sciences, Institute of Psychology, University of Gdańsk, Gdańsk, Poland

**Keywords:** physiotherapy, physical activity, cancer, mental disorders, rehabilitation

## Abstract

Physiotherapy in oncology is a process closely related to cancer treatment methods. Rehabilitation is based on physical activity in various forms involving the musculoskeletal system but also affects the emotional state. Physical activity influences physical and psychological well-being of people undergoing oncological treatment, in the course of which the most common psychiatric disorders are depression, substance use disorder, sleep disorders, fatigue syndrome, resulting in worsening of the quality of life. Difficulties in implementing physical exercise in cancer patients pose a challenge to treatment teams.

## Introduction

Physiotherapy in oncology is a process closely related to cancer treatment methods. The main tool for the improvement of people with cancer is physical activity in all possible forms, both in passive and active involvement of the motor system. The term “motor activity,” is used in the world information system as “physical activity.” It is a component of a healthy lifestyle. Without it, any health strategy, its maintenance and multiplication is impossible, and in case of children, moreover, a proper development. Physical activity is a basic determinant of fitness and physical efficiency, which are also measures of health.

Both in healthy and sick people, physical activity significantly influences the level of physical performance and is an integral component of a healthy lifestyle, therefore it must be included in the strategy of maintaining health, understood not only as the absence of disease, but full physical, mental and social well-being. This fits in with the WHO (1) definition of mental health, according to which it is a state in which a person realizes their abilities and is able to cope with a variety of life situations, can participate in social life and is able to work productively.

We know that physical activity improves not only the somatic state but also the mental state of patients. Epidemiological studies (2) show that people who start exercising or stay active are less likely to develop depression. Paffenbarger et al. observed in male alumni from Harvard College, aged 35–74 years, a reduced risk of being susceptible to depression in those with high physical activity, e.g., >2,500 kcal burned per week (relative risk of depression 0.72) and in those doing moderate physical activity e.g., 1,000–2,000 kcal burned per week (relative risk of depression 0.83), compared to those with low physical activity, considered as energy expenditure below 4.5 MET metabolic equivalent of task (relative risk of depression 1.0) (3).

Farmer et al. in an 8-year follow-up, found a 2-fold increased risk of clinical depression in women with low physical activity. Furthermore, physical activity not only reduces the risk of illness/relapse, but can also be considered a form of therapy for depression (4). This is supported by Biddle and Mutrie's (5), Stanton and Reaburn (6) review article and Craft et al. meta-analysis (7). Results from 30 studies showed that people who exercise were less depressed than individuals who did not exercise (overall mean effect–0.72). Experimental studies show that both aerobic and resistance exercise are effective in treating depression, and the effect of aerobic training is comparable to psychotherapeutic interventions (5).

The use of various supportive forms of therapy is particularly important in oncology. Cancer treatment methods—conservative, surgical and radiotherapy—are the cause of complications such as scars that limit tissue mobility, contractures, joint mobility disorders, statics disorders, swelling and a number of other complaints related to the course of therapy. These limitations affect functional impairment, reduce quality of life, and consequently the psychological state. Patients experience depression, grief, anger, rage, feelings of inferiority, guilt, and anxiety. At the root of these reactions is severe stress created by the diagnosis of the disease, uncertainty about the prognosis, inadequate social support, poor relationships with medical staff, and the impact of traumatizing life events. Young age, loss of independence, coherence disorders, sense of life threat, physical pain, medical interventions, lack of control over the course of the disease, hospitalizations, changes in physical appearance additionally increase the risk of developing depression in oncology patients and influence its course (8, 9).

The mere suspicion of cancer is a difficult fact for patients to accept. Also the highly stressful process of diagnosis, implementation of treatment and the course of therapy may be the reason for mental disorders. The most frequent mental disorders occurring in the course of cancer treatment are depressive and anxiety disorders, sleep disorders, fatigue syndrome and reduced quality of life. Therefore, it seems significant to diagnose them early in oncological patients and to provide psychological support while taking into account the necessity of comprehensive oncological rehabilitation. Psychophysical aspects of patients' improvement at every stage of oncological treatment should be carried out in cooperation between a psychologist and a physiotherapist, and implementation of broadly defined rehabilitation in patients during chemotherapy, radiotherapy and surgical treatment at all stages of cancer is a standard of treatment management. As already mentioned, a significant role can be played by physical activity already at the stage of cancer prevention and, above all, supporting the process of cancer treatment. The use of movement during rehabilitation supports cured patients in returning to normal functioning.

## Difficulties in Incorporating Physical Activity in Patients With Cancer

With numerous counter-indications to the use of physical methods in cancer, physical activity is the recommended form of physiotherapy in various types of cancer, including advanced stages of the diseases (10).

However, it is often difficult to implement and accept systematic physical activity in treated patients. This problem was described by Frawley et al. (11) in patients who underwent surgery for abdominal and pelvic cancers (including colorectal cancer, prostate cancer, and genital cancers) and were not very willing to engage in rehabilitation programs (of 84% people who were qualified to participate in the program, only 24% completed it). On the other hand, those who decided to participate in rehabilitation programs achieved beneficial therapeutic effects assessed in terms of satisfaction with participation in the program, improvement in functional ability and muscle strength, reduction in pelvic floor complaints, and level of physical activity. This group also had reduced levels of anxiety and depression and improved quality of life (*p* < 0.05).

In general, poor physical health, depressive symptoms, and lower health-related quality of life (HRQL) are associated with decreased physical activity. It is sometimes difficult to determine whether poor general health causes aversion to physical activity or whether lack of physical activity is the cause of poor general health (12).

Among the reasons for difficulty in engaging in physical activity, we can mention poor well-being associated with side effects of cancer treatment and cancer-related fatigue (CRF) syndrome. CRF, defined as the inability to exert both physical and intellectual effort, has a multifactorial etiology and considerable individual variability. It differs from typical fatigue in its degree of severity. It is described as weakness and lack of energy, impaired attention, causes difficulty in performing even a small physical effort. It is usually accompanied by apathy, which is characterized by an inability to engage in mental activity and a rapidly occurring feeling of fatigue and discouragement. Unlike typical fatigue, it does not subside with rest and sleep. Despite some similarities to depression, it is experienced as a separate condition, causing a drop in mood. It persists during treatment, but in some cases also after treatment has ended. Although no gold standard treatment is currently available for this syndrome, randomized controlled trials have shown beneficial effects of physical activity compared with control in reducing fatigue, with a mean effect size of −0.27 (13, 14).

Although physiotherapy has a documented positive effect on both the somatic and psychological recovery of people treated for cancer (15, 16) difficulties with exercise tolerance, including on the cardiovascular side, must be anticipated.

Mikkelsen et al. (17) describe that despite positive perceptions of physical activity among older cancer patients, there were problems with maintaining physical activity during cancer treatment. Factors related to cancer and aging were identified as barriers, with overwhelming feelings of fatigue being the most difficult to overcome. Improved overall physical and mental well-being, a set rhythm of activities (e.g., exercise and group supervision), and social support were identified as motivators and facilitators of activity. Preferences for forms of physical activity varied, but familiar activities increased motivation. Exercise programs for patients with cancer must be tailored to each patient's limitations, needs, and personal resources (18).

A similar study by Frikkel et al. (19) noted that weakness due to cancer therapy was the most commonly reported barrier to physical activity among both physically active and inactive patients. They examined 141 of 440 eligible patients (32.0%) with various oncologic diagnoses. The most common barriers to physical activity cited by patients were weakness due to cancer therapy (76.6% of individuals), fatigue/insomnia (71.6%), and functional impairment due to cancer therapy-related fatigue as measured by the Functional Assessment of Cancer Therapy Fatigue (FACT-F) questionnaire (70.2%). After statistical analysis, fatigue and clinically significant depression were found to be negative predictors of exercise motivation. Patients who were physically active prior to their illness and those who were interested in exercise and who believed that exercise would improve their quality of life were more motivated to exercise. Motivated patients were 5.6 times more likely to be physically active (*p* < 0.001).

It is worth noticing that the mental state is closely related to the motivation of patients to engage in therapy, including physiotherapeutic activities. Experiencing the fact of a potentially life-threatening disease may become the cause of adaptation disorders of a depressive-anxiety type. Insomnia, anxiety, fatigue caused by cancer are risk factors for depression, but on the other hand they may already be symptoms of depression. A meta-analysis of 27 studies on sleep disorders in women with breast cancer indicates that the frequency of these disorders (insomnia, problems falling asleep and waking up during the night) showed a pooled estimate of 0.40 [95% confidence interval (CI) = [0.29–0.52], *I*^2^ = 100%, *p* < 0.00001] and ranged from 0.14 (95% CI = [0.04–0.24]) to 0.93 (95% CI = [0.91–0.95]). Pain, hot flashes, non-Caucasian race and menopausal period, especially depressive symptoms and fatigue, were statistically significantly associated with the occurrence of sleep disorders (20).

Motivating to be active during and after cancer treatment is an interdisciplinary task for both medical staff and patients' families. Non-medical predictors such as the location of the rehabilitation center, experiences with previous physical activity, and the level of difficulty of the applied physical tasks are also mobilizing factors. These factors should be considered when developing and implementing exercise interventions (21, 22).

## Benefits of Physical Activity on the Mental Health of Oncology Patients

Planned and deliberate physical activity affects psychological well-being because it is inherently oriented toward health and high quality of life (23).

In particular, physical activity, understood as any kind of body movement induced by working muscles and causing an energy expenditure exceeding the resting energy level, plays a significant role in primary prevention and is a complement to therapy based on established and traditional methods of treating depressive disorders. In cancer, prevention and effective treatment of depression can significantly affect the prognosis of the underlying disease (24).

The most common side effects of oncological treatment are disorders of the locomotor system (reduced joint mobility, paresis and muscle paralysis), reduced efficiency of the circulatory system, lymphedema, reduced efficiency of the respiratory system, e.g., due to impaired lung ventilation. The range of physiotherapeutic actions in such cases can be preventive, curative or palliative. These activities include preventing complications and functional consequences of treatment, alleviating symptoms of terminal illness and improving quality of life. Physical activity can potentially counteract a number of side effects of chemotherapy or hormonal therapy, such as fatigue syndrome, weight gain, muscle atrophy, hot flashes, nausea, or increased susceptibility to infection.

We know that physical activity can be therapeutic for individuals with severe mental illness who generally have sedentary lifestyles and experience numerous lifestyle-related medical complications. On a physiological level, regular exercise can potentially increase levels of serotonin, brain-derived neurotrophic factor (BDNF), and endothelial growth factor (VEGF) (25).

Appropriately selected regular physical activity can be a form of prevention of depressive disorders. It should be treated as a technique supplementing pharmacological and psychotherapeutic treatment of depressive disorders, including those occurring in the course of cancer. This is the opinion of the European Psychiatric Association supported by the International Organization of Physical Therapists in Mental Health (IOPTMH) concerning physical activity as a method of treating serious mental illnesses (26).

On a psychological level, physical training can improve mood, self-esteem, and reduce anxiety and pain in patients. The relationship between improvements in self-reported physical status and improvements in overall self-esteem, depression, and anxiety confirms the role of the physical concept of self in the recovery of psychiatric patients with depression and anxiety and other syndromes (26–33). In depressed adults, compared to control group, sensitivity analyses revealed a moderate to large effect in favor of endurance exercise [SMD: −0.79 (90% CI: −1.10, −0.48); *p* < 0.00001, *I*^2^ = 84%] and a large effect size in favor of neuromuscular exercise [SMD: −1.14 (90 CI: −1.50, −0.78); *p* < 0.00001, *I*^2^ = 80%] (33).

When recommending physical activity to people suffering from depression, it is essential to consider that inadequate motivation may activate feelings of guilt, suicidal thoughts and behaviors. Therefore, both as a form of primary prevention and as a method complementary to pharmacotherapy and psychotherapy, it should be tailored to the individual capabilities and health status of the trainees in terms of intensity, duration, and frequency (24).

Despite ongoing research, there are no clear recommendations for the use of different forms of physiotherapy and physical activity in different types of cancer.

Physical treatments (massage, electrotherapy, hydrotherapy, and others) in oncology patients are one of the most controversial topics in rehabilitation planning. Cancer is cited as a counter-indication to their use. The development of imaging techniques puts this issue in a new light. Based on the evidence, most articles (34) report beneficial effects of physiotherapy in oncology patients, and only a few list it as harmful. Of course, each patient requires individual assessment, but if we exclude the possibility of relapse and metastasis then most physiotherapy interventions can be used safely.

The relationship between physical activity, psychological well-being and better cancer treatment is very clear. For example, among the recommended non-pharmacological treatments for depression in breast cancer patients, yoga with meditation is mentioned alongside psychotherapy as reducing depressive symptoms in these patients. However, more research is needed to determine the magnitude of this effect, depending on the severity of depression and the presence of co-existing diseases (35).

Grégoire et al. (36) compared the effectiveness of interventions based on cognitive behavioral therapy (CBT), self-hypnosis, and yoga in people with breast cancer. Nine months after the intervention, a decrease in anxiety (*p* = 0.000), depression (*p* = 0.000), and fatigue (*p* = 0.002) was observed in the hypnosis group and a decrease in anxiety (*p* = 0.024) in the yoga group, while no change in the studied parameters was found in the CBT group and the control group without any of these interventions. The combination of different physiotherapeutic approaches seems to be an interesting psychological approach to improve the well-being of patients with breast cancer. However, further research is needed to better understand the mechanisms of such interventions and their long-term impact on quality of life.

Exercises based on yoga, can be used as a form of physical activity. The work of the musculoskeletal, circulatory and respiratory organs during practice is evident. Studies (37–39) confirm the positive effects of dynamic yoga (hatha yoga) and related meditation or pranayama practices on health, including the treatment of depression, insomnia, and psychosomatic disorders. Chronic inflammation may fuel declines in physical function leading to frailty and disability.

Although the mechanisms of the observed improvements in mental health are not yet fully understood, yoga techniques can be used as an addition to pharmacotherapy. Given the growing awareness of the impact of lifestyle, stress reduction, and the importance of moderate exercise for health, various relaxation and movement techniques may complement therapies used for most psychiatric disorders. No negative side effects of supplementing standard therapies with exercises based on various forms of yoga have been demonstrated. If yoga dampens or limits both fatigue and inflammation, then regular practice could have substantial health benefits (40).

Another study compared five areas of rehabilitation after breast cancer treatment, including exercise and physical activity, complementary and alternative medicine, yoga, lymphedema management, and psychosocial interventions. Clear evidence of effectiveness was found for exercise and yoga. Exercise interventions improved shoulder mobility, reduced lymphedema, pain, fatigue, and improved quality of life. In contrast, yoga improved quality of life, reduced anxiety, depression, sleep disturbances, fatigue, and gastrointestinal symptoms. The effects of complementary and alternative medicine on nausea, pain, fatigue, anger, and anxiety were demonstrated, but these results should be interpreted with caution due to the poor methodological quality of the studies included in the evaluation (35). Physical activity also improves the overall health of people with cancer, thereby facilitating treatment. This is evidenced by the Leach et al. (41) study, which showed that physical inactivity was associated with more comorbidities following a cancer diagnosis.

According to a meta-analysis by Zeng et al. (42) (10 studies, 838 participants), in cancer patients undergoing chemotherapy, exercise has beneficial effects on physical performance and reduces depression.

One of the problems affecting people undergoing cancer treatment is an aversion to social interaction due to poor psychological well-being. Brand et al. (43) showed that in 129 people with psychiatric disorders, even one session of exercise increased interest in social contact and interaction.

The attractiveness of forms of exercise seems to be a key factor in encouraging people to undertake it. Nordic walking (NW) seems to be an interesting rehabilitation method for women with breast cancer. Sánchez-Lastra et al. (44) conducted a study that analyzed the effects of NW on women with breast cancer. The results of the studies analyzed indicated that NW had a significant and positive effect on a number of breast cancer symptoms, including lymphedema, physical performance, disability, and sense of illness. No side effects were reported.

Another form of exercise worth recommending is Pilates. It is a system of harmonious, slow exercises involving all muscle groups, combined with breathing exercises. Eyigor et al. (45) conducted a study on the effects of Pilates on functional capacity, fatigue, depression, and quality of life in 60 women, ages 18 to 75, with breast cancer. Patients who performed Pilates and home exercises (group 1) and patients who performed only home exercises (group 2) were compared. Subjects were evaluated before and after the rehabilitation program and it was found that in group 1, scores on the 6-minute walk test (6MWT), Beck Depression Inventory (BDI), and quality of life as measured by the EORTC QLQ-C30 and EORTC QLQ BR23 improved (*P* < 0.05). In group 2, there was no significant improvement in the parameters compared with the pre-exercise period. When the two exercise groups were compared, there were significant differences in 6MWT in the pilates exercise group (*p* < 0.05).

Physical activity is not only important in post-treatment rehabilitation, but is also part of the process of cancer therapy and prevention of cancer reoccurrence. In fact, physical activity after treatment for breast cancer, colorectal cancer and prostate cancer has been shown to reduce the risk of recurrence of the disease (46).

Movement is a manifestation of human physical and mental health. Therefore, activating patients only in illness is a bad strategy. The concept of lifelong activity should also include the period after recovery from cancer. Successful cancer treatment and longer life of cured patients require maintenance of physical fitness and quality of life. Physical activity is a good and inexpensive way to address physical and psychological problems. A meta-analysis by Saskija et al. (47) examined the results of 56 studies evaluating the effects of behavioral techniques and exercise on fatigue, depression, anxiety, body image, stress, and quality of life in breast cancer patients during treatment and after recovery. Statistically significant results were found for the effects of behavioral techniques on fatigue (*p* < 0.001), depression (*p* < 0.001), anxiety (*p* < 0.001), and stress (*p* = 0.038). Statistically significant results were found for the effects of exercise on fatigue (*p* = 0.004), depression (*p* = 0.016), body image (*p* = 0.007), and quality of life (*p* = 0.001).

This is supported by the results of the multicenter, randomized phase III BREX (48) study on the effectiveness of exercise in preventing long-term side effects of complementary treatment and breast cancer reoccurrence in women (*n* = 444). The purpose of the study was to investigate whether regular exercise can reduce the long-term side effects of complementary treatment for breast cancer and improve quality of life. Women aged 35–68 years who had completed complementary chemotherapy or started hormonal therapy for breast cancer within the previous 4 months participated in the study. Physical activity levels were assessed by diary, physical fitness by a 2-km walk test, quality of life by the EORTC QLQC30 and BR-23 questionnaires, fatigue by the FACIT-fatigue scale, and depression by the Beck Depression Inventory (BDI) 13-item scale. Participants who improved their level of physical activity over the 5-year follow-up were more likely to improve their global health score (*p* = 0.016), physical (*p* = 0.009), social (*p* = 0.013), role functioning (*p* = 0.005), and fatigue (*p* = 0.002). Better performance on the 2-km walk test was associated with improvements in global health, physical and role functioning, body image, future outlook, and fatigue (*p* = 0.011, *p* < 0.001, *p* = 0.001, *p* = 0.021, *p* = 0.012, *p* = 0.003).

The results obtained allow us to claim that improving the level of physical activity or physical fitness causes a positive change in the quality of life of patients with breast cancer.

A study conducted by Adams et al. (49, 50) on 63 male testicular cancer survivors (TCS) confirmed the importance of physical training in improving the mental state of patients diagnosed with cancer. These subjects were treated with 12 weeks of high-intensity interval training (HIIT). Participant-reported changes including cancer-related fatigue CRF, depression, anxiety, stress, self-esteem, sleep quality, and health-related quality of life were observed. Status was assessed at the beginning, after the intervention, and at 3-month follow-up. Cardiorespiratory fitness was also examined as a mediator of intervention effects. This effect size is larger than the 0.22–0.30 reported in recent meta-analyses of aerobic exercise and fatigue in cancer survivors (50). Statistical analysis revealed that training significantly reduced CRF (*p* = 0.003), improved self-esteem (*p* = 0.029) and multiple HRQoL domains (*p* ≤ 0.05) compared to subjects without such training. Effects on CRF (*p* = 0.031) and vitality (*p* = 0.015) persisted after 3 months of follow-up. Changes in cardiorespiratory fitness mediated improvements in CRF and HRQoL. Improvements in CRF were greater for TCS with inactive lifestyles, lower fitness, higher testosterone, and clinical fatigue at the beginning (49, 50).

Great problem in physiotherapy is the inconsistency of physical parameters applied such as doses of stimuli used, frequency and duration of treatments. In contrast to the various recommendations for physiotherapy interventions in patients with cancer, physical activity is a commonly recommended factor that is not questionable and affects mental and physical well-being. So how can such activity best be tailored to the patient's condition, taking into account their mental health? Future research should incorporate mediator analysis, biomarker testing, use appropriate control and comparison groups, evaluate outcomes using psychometric measures, and prioritize pragmatic trials toward moving into routine practice. Schuch et al. (51) makes a point to consider the relationship between potential biological mediators (e.g., BDNF) and exercise intensity as well as duration when designing exercise interventions. Therefore, developing a typology to match the most appropriate exercise prescription to the “type” of depression would both help advance research and facilitate clinical application. In studying the effectiveness of exercise programs, it is important to remember that the use of alternative exercise methods as a “control” creates a serious difficulty—usually these alternative methods also have antidepressant effects.

Psychometric scales that can accurately reflect changes in mild to moderate levels of depressive symptomatology should be favored in assessing outcomes, as these are likely to be the most common levels of symptom severity in selected samples. Although, when assessing the effects of rehabilitation in everyday physiotherapy practice, it is worth noting that in people with mild to moderate depression, a small change in symptom severity may be difficult to discern without the use of objective methods such as psychometric scales.

Further research will allow specific methods for combining non-pharmacological therapies to promote restoration of lost function during cancer and cancer prevention to be devised (52).

## Physical Activity in Cancer Prevention

In order to maintain health, systematic activity and appropriate levels of activity are essential. Determining the optimal level of physical activity is completely individualized. It depends on living conditions, gender, health status, fitness level and genetic factors. The optimum of activity to maintain health is different from that to improve health. AF directly or indirectly affects the other components of lifestyle. People who exercise regularly tend to smoke less, consume less alcohol, sleep better and eat better, which reduces the risk of cancer and increases life expectancy (53).

Associations of low physical activity with increased risk of many types of cancer are documented. This is especially true for breast, colorectal, and endometrial cancers. Physical activity also likely contributes to a lower risk of prostate and lung cancer. The largest body of research, however, concerns breast cancer. A meta-analysis of 47 out of 62 studies found that physical activity reduces breast cancer risk by 25–30% in pre- and postmenopausal women (54).

Many scientific societies and organizations from the Center for Disease Control, the National Cancer Institute, and the American Cancer Society (ACS) dedicated to cancer prevention and treatment have reported^1^ (55, 56) that physical activity is associated with or may be associated with a lower risk of cancer. Whether this is due to physical activity alone is unknown, but it undoubtedly has beneficial effects on other cancer risk factors.

The American Cancer Society has published guidelines (57) on diet and physical activity in cancer prevention in 2020.

Physical activity levels for adults should be 150–300 min of moderate-intensity physical activity per week or 75–150 min of high-intensity physical activity or an equivalent combination; reaching or exceeding the upper limit of 300 min is optimal. Children and adolescents should do at least 1 h each day of moderate to vigorous activity per week.

According to the ACS, it is important for cancer prevention to reduce sedentary lifestyle, lying down and watching TV and other forms of screen entertainment.

Recommended activities include exercise and recreational activities such as walking, dancing, recreational cycling, skating and roller skating, horseback riding, kayaking, yoga, jogging and running, fast cycling, strength training, swimming, skipping, aerobics, and martial arts. Recommended sports include downhill skiing, golf, volleyball, basketball, badminton, tennis, soccer, field hockey, lacrosse, and singles tennis (58). Daily activities and household chores such as mowing the lawn, working around the house and in the garden, and repair work are also mentioned as possible forms of physical activity. This also applies to activities related to professional work^2^.

## Summary

The presented information proves that physical activity is an effective method of supporting cancer treatment and plays an important role in its prevention. Individualization of physiotherapy programmes has a positive effect on patient co-operation. In order to overcome barriers, patients in advanced stages of cancer should be offered programmes that include information, motivational counseling and individualized exercise training. Collaboration with mental health professionals can also have a significant impact on patients' motivation to engage in physical activity.

It is clear that more than one intervention can have a positive effect on a particular symptom and that the effects depend not only on the type of intervention but also on how and when the intervention is delivered. Further research is needed to develop specific guidelines for the use of physical activity according to the somatic and psychological status of patients with cancer.

## Author Contributions

All authors listed have made a substantial, direct and intellectual contribution to the work, and approved it for publication.

## Funding

This study was financed by university funds, no external funds were used.

## Conflict of Interest

The authors declare that the research was conducted in the absence of any commercial or financial relationships that could be construed as a potential conflict of interest.

## Publisher's Note

All claims expressed in this article are solely those of the authors and do not necessarily represent those of their affiliated organizations, or those of the publisher, the editors and the reviewers. Any product that may be evaluated in this article, or claim that may be made by its manufacturer, is not guaranteed or endorsed by the publisher.

## Footnotes

^1^Division of Cancer Prevention and Control. Available online at: http://www.cdc.gov (accessed July 31, 2021).

^2^Available online at: https://www.cancer.org/healthy/eat-healthy-get-active/acs-guidelines-nutrition-physical-activity-cancer-prevention/guidelines.html.
